# Multi-Beat Averaging Reveals U Waves Are Ubiquitous and Standing Tall at Elevated Heart Rates Following Exercise

**DOI:** 10.3390/s20144029

**Published:** 2020-07-20

**Authors:** Marwa S. Al-Karadi, Philip Langley

**Affiliations:** Faculty of Science and Engineering, University of Hull, Kingston upon Hull HU6 7RX, UK; m.al-karadi@hull.ac.uk

**Keywords:** ECG U wave, ECG T wave, multi-beat averaging algorithm, exercise ECG, high heart rates

## Abstract

The reporting of U wave abnormalities is clinically important, but the measurement of this small electrocardiographic (ECG) feature is extremely difficult, especially in challenging recording conditions, such as stress exercise, due to contaminating noise. Furthermore, it is widely stated that ECG U waves are rarely observable at heart rates greater than 90 bpm. The aims of the study were (i) to assess the ability of multi-beat averaging to reveal the presence of U waves in ECGs contaminated by noise following exercise and (ii) to quantify the effect of exercise on U wave amplitude. The multi-beat averaging algorithm was applied to recover U waves in 20 healthy subjects in pre- and post-exercise recordings. Average beats were generated from 30 beat epochs. The prevalence of U waves and their amplitudes were measured in pre- and post-exercise recordings and changes in amplitude due to exercise were quantified. U waves were present in all subjects in pre-exercise recordings. Following exercise, U waves could not be seen in standard ECG but were observable in all 20 subjects by multi-beat averaging and despite significantly increased mean (±SD) heart rate (63 ± 8 bpm vs. 100 ± 9 bpm, *p* < 0.0001). Furthermore, U waves were observable in all subjects with heart rates greater than 90 bpm. U waves significantly increased in amplitude following exercise (38 ± 15 μV vs. 80 ± 48 μV, *p* = 0.0005). Multi-beat averaging is effective at recovering U waves contaminated by noise due to exercise. U waves were measurable in all subjects, dispelling the myth that U waves are rarely seen at elevated heart rates. U waves exhibit increased amplitudes at elevated heart rates following exercise.

## 1. Introduction

U waves are a small amplitude feature of the electrocardiogram (ECG) occurring at the end of the T wave [1,2,3]. Their genesis is uncertain; therefore they remain a focus of mechanistic research [1,2,3]. They are clinically important, and the major cardiac organisations, such as the American Heart Association, the American College of Cardiology, and the Heart Rhythm Society recommended that U wave abnormalities be reported during ECG interpretation [2,3]. U wave abnormalities, such as large or inverted U waves, are linked with a range of cardiac diseases, such as ischemia, hypokalaemia, and hypertension, and medications and exercise may also affect U waves [1,2,3,4].

U waves are difficult to measure due to their low amplitude, and contaminating noise or other ECG features may often obscure them. For example, U waves are obscured by the atrial fibrillatory wave during atrial fibrillation [5]. Similarly, at high heart rates the proceeding P wave may obscure the U wave [6]. During stress exercise, ECGs are often contaminated by considerable artefacts due to muscle noise, degraded skin-electrode contact, and respiratory and movement noise further impairing the ability to accurately detect and measure U waves at elevated heart rates. Hence, little is known about U waves at elevated heart rates following exercise.

Furthermore, it is commonly stated that U waves cannot be seen at modestly increased heart rates. Surawicz stated that U waves could be observed in 90% of cases when the heart rate is less than 65 bpm, while they are observed in only 25% of cases when the heart rate is 80 to 90 bpm, and are rarely detectable when the heart rate exceeds 95 bpm [1]. Other authors have reasserted this claim [6,7,8]. It is unclear if the reason for not being able to detect U waves at high heart rates was due to mechanistic reasons (i.e., U waves are not generated at high heart rates) or if they were undetectable due to technical reasons, such as being obscured by contaminating noise or P waves of the following beat. Therefore, the aim of our study was to investigate the prevalence and amplitude of U waves at elevated heart rates induced by exercise in healthy subjects with due regard to contaminating noise and proceeding P waves using a multi-beat averaging algorithm.

## 2. Materials and Methods 

### 2.1. ECG Recordings 

The recordings were 12-lead ECGs from 20 healthy subjects (15 men; age 34 ± 10 years, with no history or symptoms of heart disease) obtained while following a resting, exercise, and post-exercise recovery protocol. These were historical recordings that had been used for previous research studies [9]. The participants gave informed consent at the time of recording. The study was conducted in accordance with the Declaration of Helsinki and approved by the Ethics Committee of the Faculty of Science and Engineering at the University of Hull (FEC_2019_111).

**Figure 1:**
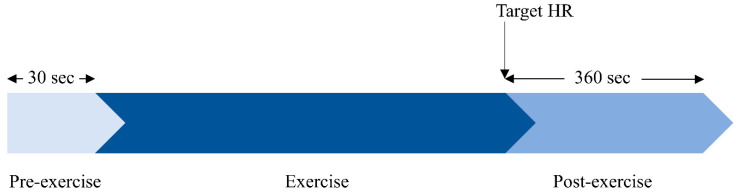
Study protocol showing pre-exercise, exercise and post-exercise phases.

For each subject the protocol comprised three distinct phases: a pre-exercise resting phase followed by an exercising phase and subsequent post-exercise recovery phase. Following the 30 s supine resting phase the subjects underwent upright treadmill exercise to achieve their age-adjusted maximum heart rate using the Bruce protocol [9]. Immediately following exercise and after reaching the specified heart rate, subjects returned to the supine position and post-exercise recovery was recorded for 360 s. The exercise phase had variable duration according to the subjects’ cardiovascular response to the exercise and target heart rate. The protocol is illustrated in Figure 1.

ECG leads were recorded to a computer via a bioelectric amplifier (gain 1000, bandwidth 0.05 to 100 Hz at a sample rate of 500 Hz with 4.9 µV resolution). Because the U wave has the largest amplitude in the precordial leads [3,10] V4 was analysed except in the case of poor electrode contact in that lead, in which case V2 was analysed (Table 1). 

### 2.2. Beat Averaging Algorithm to Recover U Waves

Exercise ECG is usually contaminated by noise, making it difficult to observe small amplitude features such as U waves. In our effort to facilitate the measurement of U waves in challenging conditions we have previously demonstrated ventricular beat averaging as an effective tool to reveal U waves when they are completely obscured by the fibrillatory wave in atrial fibrillation [5]. We hypothesise that the same algorithm can be applied to recover U waves from ECGs contaminated by noise due to exercise. 

The algorithm uses signal averaging, which is a well-known signal processing technique used extensively to reduce noise when multiple repetitions of a small amplitude signal are available for measurement [5,11,12]. It allows small amplitude signal components to be measured in the presence of large amplitude noise, but only when the signal and noise are uncorrelated [11,12,13]. By careful alignment and averaging of increasing numbers of ventricular beats, the amplitude of the uncorrelated noise is progressively reduced while the desired U wave is revealed in the resulting average beat. The level of noise reduction achieved is a function of the number of averaged signal epochs (*N*) where it can be reduced by a factor of √*N* [5,11,12,13]. 

**Figure 2:**
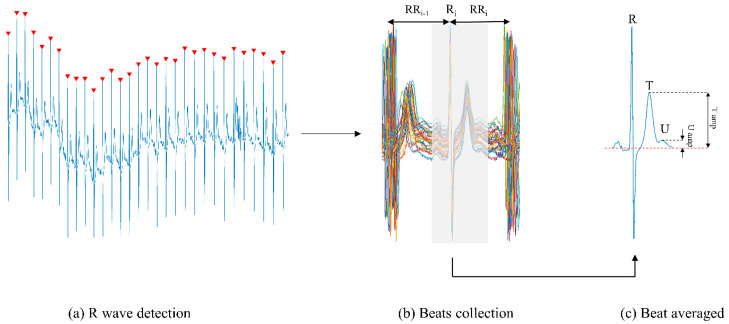
Electrocardiogram (ECG) signal processing stages to recover the U wave illustrated in a post-exercise recording. (**a**) R wave peaks (▼) were detected in the ECG lead. (**b**) Qualifying beats were collected and aligned to their R peak (R_i_). Beats with RR_i_ < 500 ms were excluded to avoid U wave contamination from following P waves. An averaged beat was calculated by averaging the collected beats in a window comprising the P wave, QRS complex, T wave and U wave (represented by the grey shaded area). (**c**) The U wave is readily observable in the average beat and the amplitude of the U wave (U amp) was measured from the stable baseline (red dashed line) to the peak of the U wave. T wave amplitude (T amp) was also measured.

The method of generation of an averaged beat is illustrated in Figure 2. First, R wave peaks are detected automatically and inspected visually to confirm their accurate detection (Figure 2a). Second, the qualifying beats are collected and aligned to their R wave peaks (indicated R_i_ in Figure 2b). Qualifying beats are those that do not have P waves encroaching the U wave. To meet this condition, we set a requirement that beats included for beat averaging must have a following beat interval (RR interval) (indicated as RR_i_ in Figure 2b) greater than 500 ms. The collection of beats was plotted to confirm visually that P waves did not contaminate the U waves (Figure 2b). The beat collection was also inspected for ectopic beats, but none were present in any of the recordings. Following beat collection, the average beat was calculated as the average across each sample point (Figure 2c). Figure 2c shows that U waves could readily be observed in the average beat. 

The number of beats included in the formation of the average beat is an important consideration because, as discussed above, it determines the extent of noise reduction. We observed that in all subjects 30 beats were sufficient to provide clean U waves. Hence for each subject the first consecutive 30 beats meeting the qualifying beat criterion were collected to generate the averaged beat in both pre- and post-exercise recordings. 

### 2.3. Measurement of U and T Wave Amplitudes

U wave amplitudes were automatically measured from the average beats of the pre- and post-exercise recordings. The amplitudes were measured from a stable baseline to the U wave peaks (Figure 2c). The stable baseline was estimated as the average of a 10-sample window in the electrically inactive period before the onset of the P wave. The U peak was detected by searching for the maximum amplitude within a predefined window. All automatically detected baselines and peaks were visually assessed for accuracy. 

The effect of exercise on T wave amplitudes have been previously reported [9]; however, for completeness we also measured T wave amplitudes.

### 2.4. Statistical Analysis 

The resting, pre-exercise recordings provided the reference measurements from which the changes in U and T waves observed in the post-exercise recordings were assessed. Both the pre- and post-exercise recordings were obtained in the supine position, thus eliminating any potential postural effects on the waveform features. The paired *t*-test was used to determine the significance of differences in pre- and post-exercise U and T wave amplitudes and heart rate. All processing software and statistical analysis were implemented in MATLAB 2017.

## 3. Results

### 3.1. Prevalence of U Waves in Pre- and Post-Exercise Recordings

**Figure 3:**
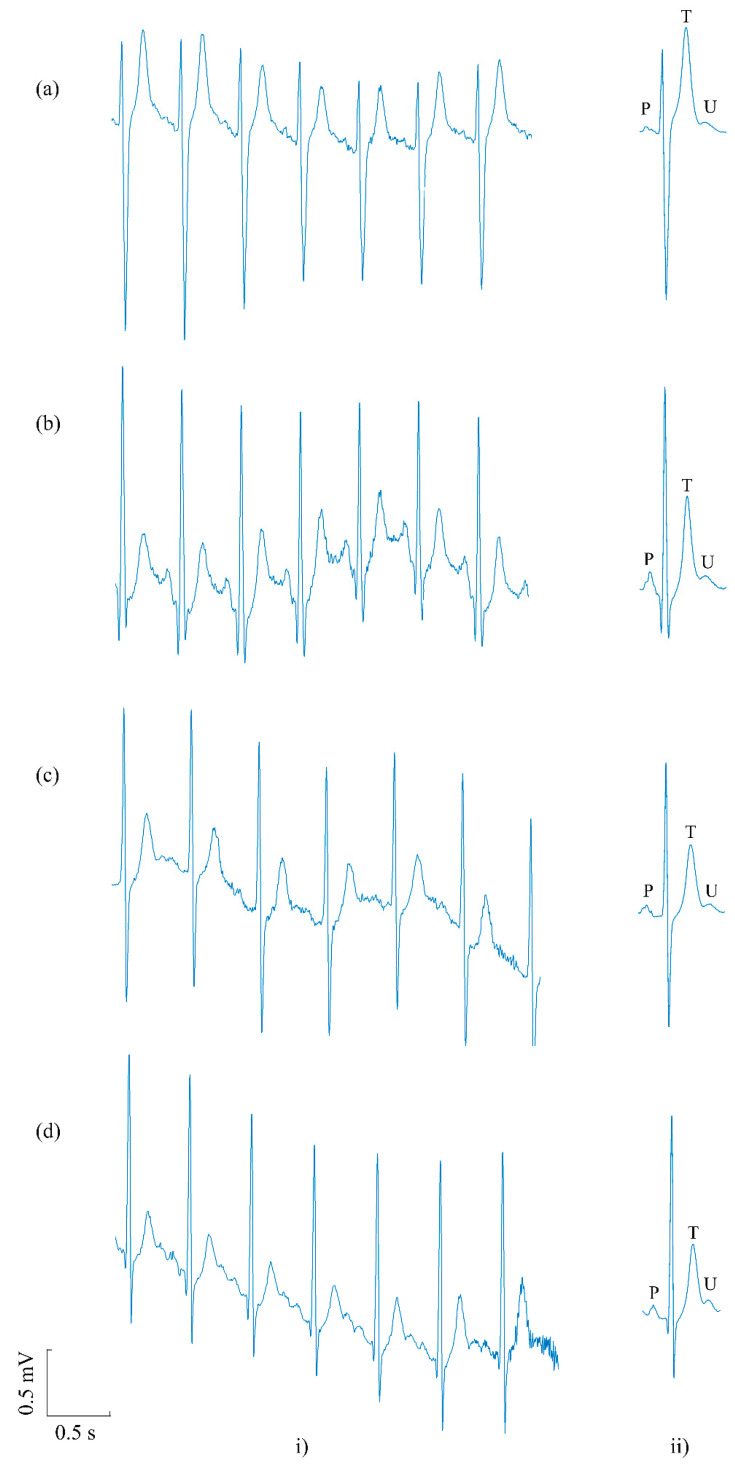
Examples illustrating the recovery of U waves in four subjects (**a**–**d**) from post-exercise recordings using the beat averaging algorithm. (**i**) A 4 s strip of post-exercise ECG in which it is difficult to recognise U waves in the four subjects. (**ii**) The corresponding averaged beat in post-exercise with a clear U wave following the T wave.

U waves were present in all subjects in pre-exercise recordings. Despite significantly increased heart rates (63 ± 8 bpm vs. 100 ± 9 bpm, *p* < 0.0001), U waves were also present in all subjects in post-exercise recordings following beat averaging. Figure 3 shows representative examples of U waves recovered from post-exercise recordings for four subjects (Figure 3a–d). The figure shows, for each subject, a 4 s strip of post-exercise ECG in which U waves cannot be seen due to the obscuring noise (Figure 3i). Also shown are the associated average beats generated by the algorithm in which clean and uncontaminated U waves can be readily observed for each subject (Figure 3ii). 

### 3.2. The Effect of Exercise on U Waves

Following exercise, U waves significantly increased in amplitude (38 ± 15 µV (mean ± SD) vs. 80 ± 48 µV, *p* = 0.0005).

**Figure 4:**
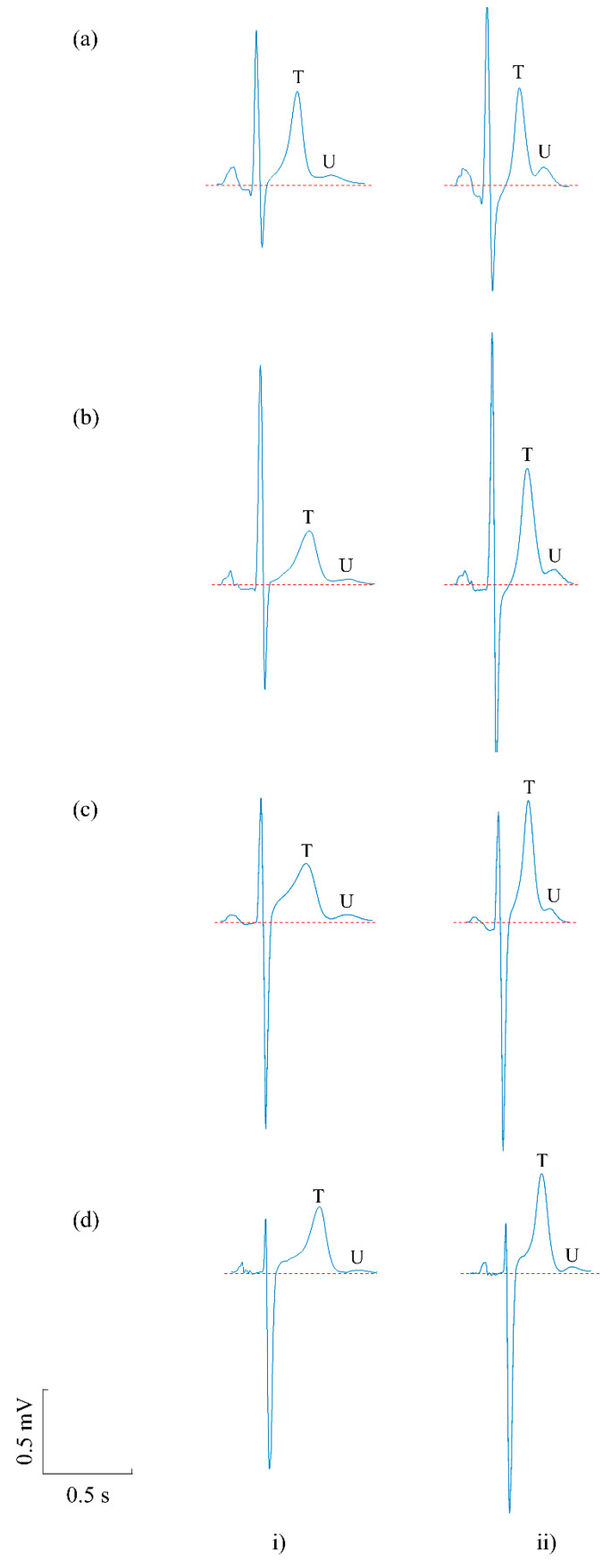
Examples comparing ECG features in (**i**) pre- and (**ii**) post-exercise recordings for four subjects (**a**–**d**). In both pre- and post-exercise recordings, U waves in the averaged beats were visible and measurable; however, U wave amplitudes were higher in the post-exercise condition in the four subjects. T wave amplitude also increased in these subjects.

**Figure 5:**
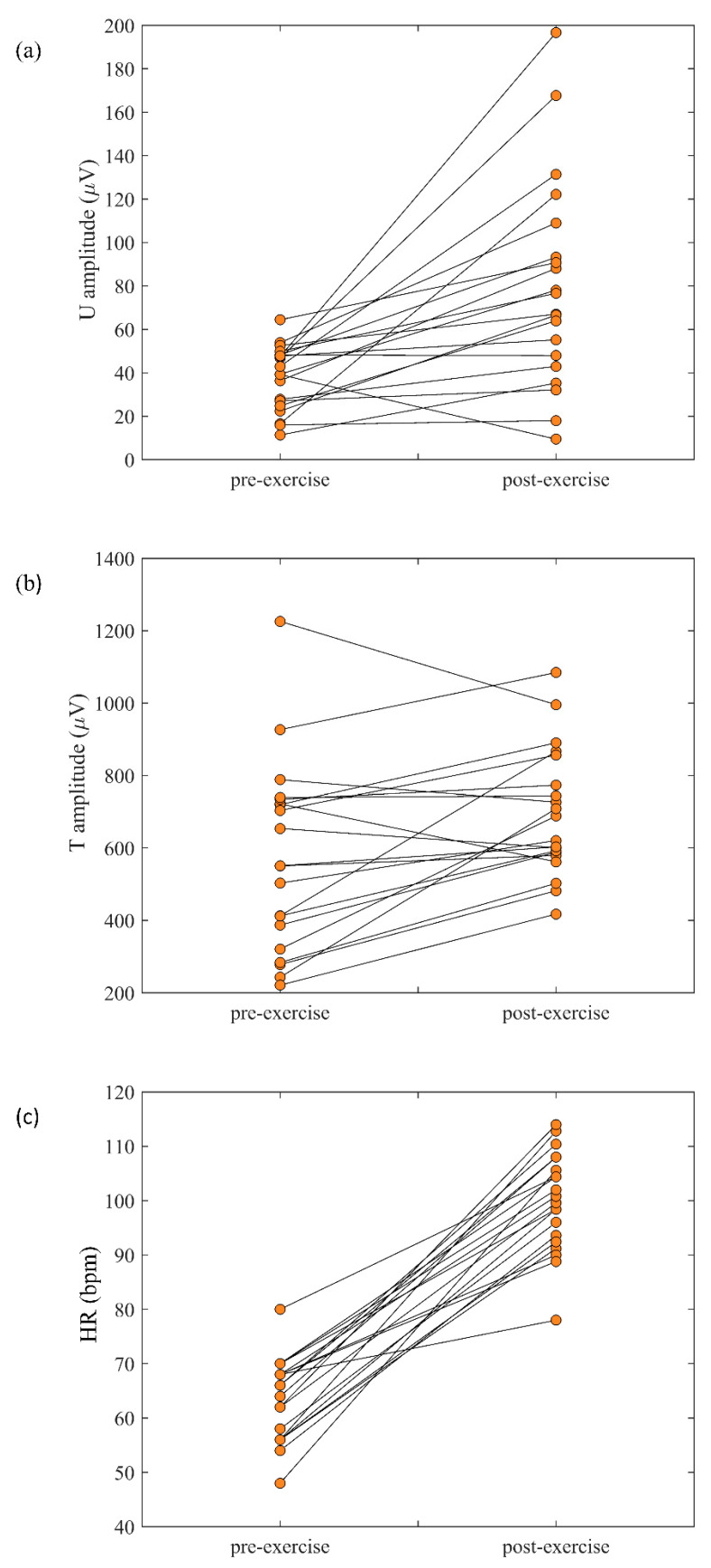
Paired relationship for (**a**) U wave amplitudes, (**b**) T wave amplitudes, and (**c**) heart rates (HR) for 20 subjects during the pre- and post-exercise recordings.

Figure 4 illustrates representative examples of the averaged beats for pre-exercise (Figure 4i) and post-exercise (Figure 4ii) recordings in four subjects (Figure 4a–d). Increases in U wave amplitude in the post-exercise recordings are clearly seen in these examples. Considering all 20 subjects, Figure 5a shows the paired pre- and post-exercise U wave amplitudes in which U wave amplitude increased in the post-exercise recordings in 18 of the 20 subjects. Measurements for individual subjects are provided in Table 1. 

Similar to the U wave, T waves showed a significant increase in amplitudes from pre- to post-exercise (569 ± 259 µV vs. 694 ± 175 µV, *p* = 0.006) (Table 1, Figure 5b). Sixteen of the 20 subjects exhibited T wave amplitude increases post-exercise.

Although there was a greater increase in T wave amplitude compared to U wave amplitude (∆T 125 ± 182 µV vs. ∆U 42 ± 45 µV, *p* = 0.0452), the increase in mean U wave amplitude represented a 111% increase from resting level compared to an only 22% increase for the T wave.

## 4. Discussion 

### 4.1. Prevalence of U Waves in Post-Exercise Recordings

Our study demonstrates unequivocally that U waves are observable at elevated heart rates. According to the literature, only very rarely can U waves be observed in subjects with heart rates above 90 bpm [1,6,7,8]. In our study, the average post-exercise heart rate was 100 bpm and 17 of 20 subjects had heart rates greater than 90 bpm, yet we could readily observe U waves in all subjects.

The U waves in post-exercise recordings were almost universally obscured by contaminating noise due to the challenging recording conditions of stress exercise ECG. Multi-beat averaging was effective at recovering the underlying U waves. Given that the mechanisms of U wave generation are still unresolved and the fact that there is high clinical value in the identification of U wave abnormalities, as expressed by the major cardiovascular organisations [1,2,3], it is important to be able to study U waves in these challenging conditions. Our study demonstrates that multi-beat averaging is a useful tool to enable the observation and analysis of U waves in such conditions.

### 4.2. The Effect of Exercise on U Waves

Having introduced a technique by which U waves can be revealed at elevated heart rates due to exercise, in this study we also provide the first quantitative analysis of the effect of exercise on U wave amplitude. The effect of exercise was to significantly increase the amplitude of U waves in the immediate post-exercise period relative to the pre-exercise resting condition. This was the case in 18 of the 20 subjects (90%) studied. Although the increase in heart rate in post-exercise recordings was relatively modest (63 ± 8 bpm vs. 100 ± 9 bpm, *p* < 0.0001), mean U wave amplitude more than doubled (38 ± 15 µV (mean ± SD) vs. 80 ± 48 µV, *p* = 0.0005), suggesting that U waves are highly dependent upon heart rate.

As expected, T wave amplitudes also increased, which agrees with a previous study using the same recordings but not using the beat averaging algorithm [9].

Given these findings, it is possible to speculate on potential mechanisms that might explain the observed effect of exercise on the important repolarisation phase of the ECG, including U waves. The mechanism of U wave genesis is still uncertain. The main hypotheses proposed to explain its genesis include: (i) delayed ventricular repolarisation of either the Purkinje system or mid-myocardial cells and (ii) stretch induced delayed after depolarisation caused by mechanoelectrical coupling [1,3,14]. According to the Frank–Starling mechanism, increases in heart rate cause increased left ventricular blood volume and accordingly more stretch to the myocytes during diastole due to increased preload [10,15]. Simultaneously, the pressure of the left ventricle increases and potentially activates the mechanosensitive ion channels, which transduce the pressure and stretch into electrical signals that modulate the underlying action potentials of the cardiac cells [15,16,17]. A previous computational study reported that heart rate changes influence the cardiac action potential such that the occurrence of after-potentials induces increased U wave amplitude [4,18]. Another plausible explanation is that exercise induces increased dispersion of repolarisation leading to both increased T wave and U wave amplitudes [19,20].

Abnormal U waves are clinically important and considered an early marker of many cardiac diseases [1,3,21]. Previous studies indicate that U wave inversion due to exercise is predictive of significant coronary artery diseases and ischemia [3,22,23,24,25,26,27]. Additionally, inverted U waves can be observed in coronary vasospasm, hypertension, left sided valvular diseases, and cardiomyopathy [3,27,28]. U wave amplitudes were reportedly increased during slow heart rates, including as a result of bradycardia, hypokalaemia, and in long QT syndrome [3,29,30], which is linked to the increase in ventricular filling due to prolonged diastole and increased ejection fraction [3,31]. As expected, we did not observe any U wave abnormalities in our study because the subjects were healthy and without a history of cardiac diseases. Our study shows that U wave amplitude increases at elevated heart rates associated with exercise.

We note that U waves and T waves did not increase in a small number of subjects, and it is worth noting the difficulty of establishing a stable baseline reference level for amplitude measurements at high heart rates. We chose the relatively stable period before the onset of the P wave as the reference level in our measurements. This was chosen because across the entire ECG waveform this is the part that is closest to quiescent. However, this part may be affected by the tail end of low amplitude residual U waves from the previous beat. However, any residual U wave present in the selected baseline would result in an elevated baseline level, which in turn would result in reduced U (or T) wave amplitude measurements relative to the true baseline. In other words, any contamination of our reference baseline levels would tend to decrease our observed effect of increased U wave amplitude, so we are confident that the observed effect is true.

The measurement technique we have described is applicable to U waves regardless of U wave morphology, be it positive, negative, or bi-phasic. It is however only valid if the waveform feature is stable and not changing significantly over the epoch considered. The algorithm, in its present implementation, would not be suitable if the U wave morphology was changing beat-by-beat, for example for the detection of repolarisation alternans.

One of the limitations of the study was that the heart rates at which U waves were measured following exercise were relatively modest, ranging from 78 to 113 bpm. The target heart rate for each subject was subject to limitation by age (age-adjusted maximum heart rate), and although subjects were then transferred rapidly to the supine position, there was a further rapid decrease in heart rate during this transfer period. On the other hand, extremely high heart rates are not beneficial for the study since the U waves become obscured by the following P waves and cannot be measured. Despite the limited heart rates achieved we have nonetheless met the two main aims of the study and shown that U waves can be observed at heart rates greater than 90 bpm and that U amplitudes are significantly increased.

## 5. Conclusions

In this study, multi-beat averaging was shown to be an effective technique for recovering U waves contaminated by noise due to exercise. Using this technique, we have demonstrated that U waves were measurable in all subjects at elevated heart rates following exercise, dispelling the myth that U waves are rarely seen at such heart rates. Furthermore, U waves exhibited significantly increased amplitudes at elevated heart rates following exercise and this observation may be explained by increased mechanoelectrical coupling, one of the major hypotheses of U wave generation. Hence our study provides mechanistic insight into U wave generation. These developments facilitate the study of U waves in challenging recordings conditions that have not previously been possible using standard ECG techniques, which in turn helps increase our understanding of U wave mechanisms and facilitates the detection of U wave abnormalities as recommended by major cardiac organisations.

## Figures and Tables

**Table 1:** Summary of 20 healthy subject measurements during pre- and post-exercise. HR = heart rate (bpm), T amp = T amplitude, and U amp = U amplitude; ∆ represents the change in amplitude between pre- and post-recordings in the respective waves.

Subject	Lead	Pre-Exercise			Post-Exercise			∆ Amplitudes (µV)	
		HR (bpm)	U Amp (µV)	T Amp (µV)	HR (bpm)	U Amp (µV)	T Amp (µV)	∆U	∆T
1	V4	70	48	654	98	48	601	0	−53
2	V2	56	48	413	98	93	867	45	454
3	V2	54	11	387	94	35	588	24	201
4	V4	64	39	789	108	78	726	39	−63
5	V4	48	47	720	106	168	890	121	170
6	V4	62	54	550	100	109	579	55	29
7	V4	56	36	321	91	88	688	52	367
8	V4	66	28	1226	110	43	996	15	−230
9	V4	70	27	278	104	32	482	5	204
10	V4	56	47	927	113	197	1085	150	158
11	V2	68	39	722	78	9	562	−30	−160
12	V2	56	64	734	92	91	774	27	40
13	V2	68	53	503	101	67	621	14	118
14	V4	66	17	413	108	122	592	105	179
15	V4	62	50	739	114	77	744	27	5
16	V4	70	48	704	102	55	857	7	153
17	V4	58	22	284	96	67	503	45	219
18	V4	80	43	551	104	131	603	88	52
19	V4	68	25	243	90	64	709	39	466
20	V4	68	16	221	89	18	418	2	197
